# Volumetric Scale-Up of a Packed-Bed Ion-Exchange System to Extract Phytate from Thin Stillage

**DOI:** 10.3390/membranes12020230

**Published:** 2022-02-17

**Authors:** Cristiano E. Rodrigues Reis, Bo Hu

**Affiliations:** 1 EARTH University, Guacimo, Limon 4442-1000, Costa Rica; crodrigues@earth.ac.cr; 2 Department of Bioproducts and Biosystems Engineering, University of Minnesota, Saint Paul, MN 55108, USA

**Keywords:** ion-exchange, scale-up, thin stillage, van Deemter

## Abstract

Phytate is the main form of phosphorus in corn ethanol coproducts and poses digestion issues in monogastric-animal feed. Extracting phytate as a commodity chemical will bring extra revenue to the corn ethanol industry and reduces potential phosphorus pollution from livestock waste management. We assessed a simplified scale-up approach of an ion-exchange separation system applied to extract phytate from thin stillage using volumetric parameters and simplifications of the van Deemter model. Thin stillage is one of the main byproducts generated on dry-grind corn-to-ethanol plants and accounts for the liquid portion of the bottom product generated in the ethanol distillation process. Thin stillage is rich in dissolved phytate, which served as the basis for an ion-exchange extraction system developed with a scalability factor of 50. Under the evaluated conditions, similar breakthrough profiles were obtained when similar Péclet and Stanton numbers were maintained for the scales studied, demonstrating that a simple and straightforward scale-up can be attained if special attention is given to maintaining both parameters as the basis of calculations of the plate numbers of ion-exchange columns.

## 1. Introduction

The largest portion of organic phosphorus in dried distiller grains with solubles is phytate. Phytate is the main storage form of phosphorus in many plant tissues in many seeds, grains, and cereals, such as corn [1]. Phytate is reported to be unavailable biologically to monogastric animals because of a lack of microbial phytases in their digestive tracts [2]. Phytate, however, has a wide range of applications in many fields due to its strong chelating properties [3,4,5].

Thin stillage is one of the main byproducts generated on dry-grind corn-to-ethanol plants and accounts for the liquid portion of the bottom product generated in the ethanol distillation process [6]. The production of animal feed from ethanol byproducts is usually overdosed with phosphorus, mainly derived from thin stillage. We previously disclosed a laboratory-scale study of extracting the main form of phosphorus from thin stillage, i.e., phytate, at high purities using ion-exchange resins [7,8,9] in order to potentially decrease the concentration of undigestible phosphorus in animal feed. The phytate removal process is not only likely to produce a value-added compound but also to significantly decrease the concentration of phosphorus in animal feed that is generated at the end of the downstream ethanol process.

Preparative chromatography is one of the main purification techniques used in the production of biological compounds, such as proteins, and pharmaceutical-grade chemicals. Chromatographic column separation depends on a variety of design and operating factors. A successful scale-up usually involves several factors being fixed on both a small and large scale, such as involved kinetic factors (e.g., particle size, pore size, temperature, mobile phase, and ligand chemistry) and dynamic variables (e.g., bed height, flow velocity, and packing density). The most common simplification when understanding chromatographic processes is expressed as the van Deemter equation [10] (Equation (1)). The van Deemter equation relates the linear flow velocity (*u*), the plate height present for a chromatographic column (*H*), and the constants for each system considered (*A*, *B*, and *C*).

(1) $$H=A+\frac{B}{u}+Cu$$

In this work, we assessed a simplified scale-up approach of an ion-exchange separation system applied to extract phytate from thin stillage using volumetric parameters and simplifications of the van Deemter model. We also addressed the anionic competitiveness of some of the anions present in thin stillage, demonstrating that the ion-exchange process is mainly controlled by phytate.

## 2. Materials and Methods

### 2.1. Experimental and Analytical Procedures

Two chromatographic columns were used with diameters of 1 and 5 cm (ACE Glass, Vineland, NJ, USA). Thin stillage was obtained from a dry-grind ethanol plant in the state of Iowa and was filtered to 0.45 µm using an ErtelAlsop 4T filter (Kingston, NY, USA). A full characterization of thin stillage was described by Reis et al. [9]. Filtered thin stillage was used as the raw material for the experiments.

Columns were packed to different bed heights with Amberlite IRA 900 resin (Sigma-Aldrich, Saint Louis, MO, USA). IRA 900 resin is characterized as a strong, basic anion exchanger with quaternary ammonium as the active exchange sites, an ion-exchange capacity of 1.0 meq mL^−1^ wet resin, a particle distribution between 0.65 and 0.82 mm, a moisture content within the range of 58% to 64%, and a density of 0.256 g mL^−1^. Some further details concerning the characterization of the resin are described elsewhere [8,11]. The packing and conditioning of the resin were adapted from He et al. [7]. Thin stillage was pumped at designated flow rates using a peristaltic pump at controlled rates. Phytate was estimated using an adapted Wade method [12]. The Wade method is based on the colorimetry of ferric ion and sulfosalicylic acid, with a maximum absorbance at 500 nm. The phosphate ester complexes with the iron, decreasing the color intensity of the Wade reagent [12]. The method was calibrated accordingly, using sodium phytate from rice (Pfaltz & Bauer, Waterbury, CT, USA) as the phytate standard. 

The breakthrough point was defined as the point in which the column efficiency was lower than 90%, i.e., the concentration of phytate at the column outlet *C* over its initial concentration *C*_0_ was greater than 10% (*C*/*C*_0_ > 0.10). Breakthrough curves were plotted using sequential sampling from the column outlet, taking the concentration of phytate as the adsorbing species and plotting it against BV.

Multicomponent isotherm experiments were performed utilizing different amounts of resin and a fixed amount of thin stillage, as described by Reis et al. [8]. Nitrate, phosphate, and sulfate were analyzed with an ion chromatography system using a Dionex AS-11 column [13].

### 2.2. Model Development

The widespread use of the van Deemter model for chromatographic scale-ups is the basis to a great share of the understanding of the application of industrial-sized chromatographic separations. Martin and Synge [14] developed the concept of the number of theoretical plates. While van Deemter’s model describes the concept of the height of a theoretical plate (*H*), Martin and Synge’s approach describes the relationship between the number of theoretical plates, *N*, to column efficiency. Considering *N = L*/*H*, *N* directly relates to the column resolution, and while maintaining column efficiency, a chromatographic system can be understood as a summation of individual plates. This is true when maintaining a similar condition of plates across a column and when possessing conditions under which the intraparticle diffusion rates are similar across the column length.

The Péclet number (*Pe*) measures the macroscopic flow dispersion in a column and is directly impacted by flow heterogeneity. For a column system, *Pe* can be measured as *Pe *=* uL*/*Da*, where *Da* is a coefficient regarding axial diffusion. Yamamoto et al. [15] derived a relationship for *Pe* in the van Deemter equation: for a fixed volumetric flow *Q* (BV per time unit), *Pe* ~ *L*/*A*. The Stanton number (*St*) describes the ratio of heat or mass transferred into a fluid and its capacity. For a resin system, it relates how the flow influences the mass transfer within a column [16]. According to Hansen [16], an approximation of 1/*CQ* ~ *St* can be made for such a system. The number of plates is an indication of separation in a packed-bed column, and the principle of scaling up is based on keeping *N* constant. Utilizing the discretion provided by the van Deemter model, *N* can be rewritten as the relationship provided by Equation (2).

(2) $$N=\frac{1}{\frac{A}{L}+CQ}$$

where *Q* (*Q* = *u*/*L*) is the column flow rate in bed volumes (*BV*) per time unit. Hansen [16] provided two major observations from the equation above: (i) for a fixed flow rate *Q*, the plate number *N* increases with the bed height; and (ii) if *A*/*L* << *CQ*, the plate number is independent of the bed height *L*. Thus, if a fixed flow rate *Q* is selected, an increase in the bed height theoretically provides similar or enhanced performance. This increase follows an asymptotic profile, meaning that after a certain column length, the increase is of low significance. If all operations are performed at a range greater than the minimum plate number, the performance should be satisfactory if *Q* is constant. Hansen [16] described such an application to a few cases of protein separation. Therefore, the hypothesis from this demonstration was that a scale-up can be developed if St and Pe are kept similar for the different scales. Further considerations are given in the Supplementary Material, also considering contributions by [10,17,18,19,20,21,22,23].

The validation of the linear scale-up herein is given as an analysis of the breakthrough curve under different experimental conditions.

## 3. Results and Discussion

### 3.1. Effect of the Column Height

**Figure 1:**
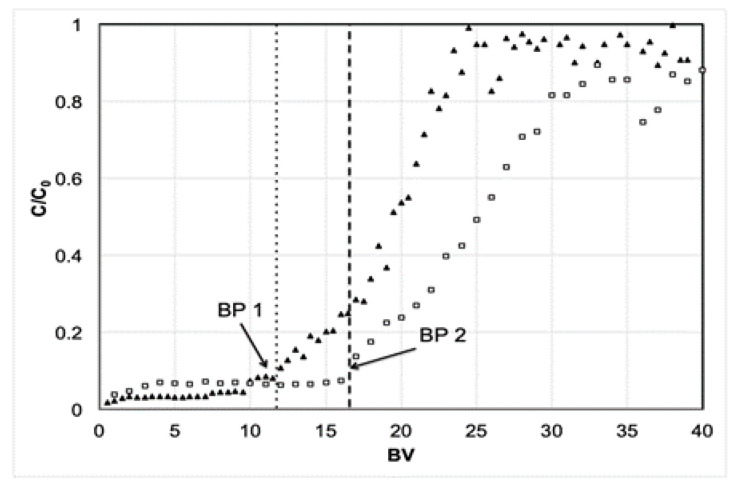
Effect of different column heights on the breakthrough curve using column heights of 2.5 cm (white squares) and 5.0 cm (black triangles).

Two runs were performed using a 1 cm packed column, one with a column height of 2.5 cm (*Q* = 2.75 *BV* h^−1^) and one with a column height of 5.0 cm (*Q* = 1.35 *BV* h^−1^), shown in Figure 1 as white squares and black triangles, respectively. Figure 1 demonstrates that the breakthrough profile of both conditions was different, represented as breakthrough point (*BP*) 1 and *BP*2, respectively. The 5.0 cm column had a sharper profile (*BP*1), which indicates that its separation efficiency increased. The 2.5 cm column, in this run, showed a wider band within the breakthrough zone. 

Two factors mentioned during the method development detailed in this paper can explain the difference, the first of which is related to the increase in *Q*, which increases *H* within a column and reduces its performance. The second factor relates to the fact that increases in column height increase *N* and may reduce *H*, which increases column performance.

### 3.2. The Effect of a Similar Q for Different Heights

**Figure 2:**
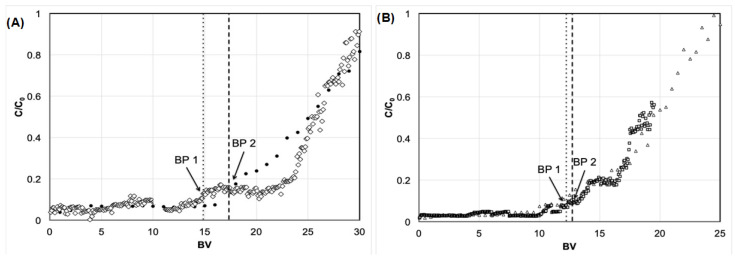
The effect of a similar *Q* on scale-up for (**A**) a high-range *Q* on the breakthrough curve considering two conditions: a 5 cm height and 5 cm diameter for the white diamonds, and a 2.5 cm height and 1 cm diameter for the black circles; and (**B**) a low-range *Q* on the breakthrough curve considering two conditions: a 10 cm height and 5 cm diameter for the white squares, and a 5.0 cm height and 1 cm diameter for the white triangles.

The results for the same *Q* (2.7 *BV* h^−1^) are depicted in Figure 2A. In this case, two different column diameters and heights were tested. The results, as expected, showed a similar breakthrough profile, as maintaining *Q* should, in accordance with Hansen [16], maintain the plate number. The results, indicated in Figure 2A for *BP*1 and *BP*2, show that the operating condition is above the minimum *Q* and height, thus indicating a linear scale-up approach based on the plate number.

Similarly, for a fixed *Q* (1.4 *BV* h^−1^) and different column heights and diameters, a sharp and coincidental breakthrough profile is presented in Figure 2B. As previously shown, a fixed *Q* above the minimum requirement for the linear scalability of plate numbers provides an easy and straightforward scale-up. However, a reduction in *Q* led to an impact on the plate height, which is indicated by a sharper breakthrough profile. Therefore, depending on the operational conditions, better performance of the column can be obtained with the presented conditions if *Q* is operated at lower levels.

### 3.3. Considerations Regarding the Scale-Up Approach for the Extraction of Phytate

The scale-up approach considers that the van Deemter model describes the mechanisms of band broadening in chromatographic separations for nonideal separations, i.e., those that do not follow a linear isotherm. For nonideal chromatography and nonlinear isotherms, which include most of the adsorption processes in mixture separations, Van Deemter et al. [17] described a comprehensive cooperation of two theories: rate theory and plate theory. Plate theory describes the separation efficiency of a chromatographic column by the height equivalent to a theoretical plate. Rate theory provides all information on the influence of kinetic phenomena, and its idealization lies within its acceptability under specific conditions, e.g., thin film cases. 

The derivation of the plate theory has been extensively described in the literature, as summarized by Xu et al. [10]. The history and elution curve of a single band in partition chromatography are taken for demonstration, and *v_i_* and *v_ii_* are defined as the volumes of the moving and immobile phase, respectively, in one theoretical plate. For a system with a distribution factor *K* that is assumed to follow a linear profile of concentrations in both phases in chromatographic separation, i.e., solid and fluid, the effective plate volume, *v*, can be defined as the relationship given as Equation (3).

(3) $$v=v_{i}+\frac{v_{ii}}{K},\;\mathrm{for}\;\mathrm{which}\;c_{i}=Kc_{ii}$$

For a system with a feed concentration equaling *c*_0_ and a feed volume equaling *A*, the material balance derived for the first plate when a volume *dS* of the fluid phase passes through is presented in Equation (4).

(4) $$v_{i}dc_{i,1}+v_{ii}dc_{ii,1}+c_{i,1}dS=\{\begin{array}{c} c_{0}dS\;for\;0\leq S\leq A\\ 0\;for\;S>A \end{array}$$

Similarly, the other plates, i.e., for *n* > 1, are described by Equation (5):

(5) $$v_{i}dc_{i,n}+v_{ii}dc_{ii,n}+c_{i,n}dS=c_{i,n-1}dS$$

The derivation of the steps in Equation (5) was expanded and mathematically demonstrated by van Deemter [17], reaching a new variable, ∆S, which is the width referent to the distance between the points of intersection of the tangents in the inflection points with the horizontal axis. Despite the complexity of the terms, both height (*c_i,n_*/*c*_0_) and width (∆S) are only dependent on *A*. In simpler terms, a straightforward approach considering the relationship between *A* and the factors involved in *Q* can be selected as the main operating drivers for a successful column scale-up. Some considerations concerning pressure drop in the column, which are not assessed in this article, are described in the Supplementary Material, based on the observations by [22,24,25,26].

Similar approaches have been described in the literature. Deshmukh et al. [27] described the potential of scaling up an ion-exchange process for the purification of oligonucleotides and touched upon key parameters involved in the process, such as temperature, organic compounds present in the buffer used in the sample separation, and binding capacity. Other reports include those published by Heuer et al. [28], who used this approach for the separation of steroids; Al-Jibbouri [29], who used it with different proteins; and Kalil et al. [30], who used it for enzyme purification. The volumetric scale-up approaches in all cases are, as highlighted by Al-Jibbouri [29], based on the guideline provided by the Van Deemter equation, in which maintaining the time scale on all conditions is a good approximation of the scaling factor of the flow rate to the effluent load to the column medium.

Considering that the recovery of phytate as a compound does not present the same economic value of pharmaceutical-grade proteins, and that thin stillage presents a complex characterization [6,9], the scale-up of the laboratory model to a potentially larger scale separation system should be as robust as possible. Though it is highly challenging to model a system with a wide variety of anions, including many of an unknown composition (i.e., fragments of charged proteins, amino acids, hemicellulose, and lignin fragments), a fundamental approach was taken to consider the scale-up of phytate extraction from thin stillage using IRA 900 resin. IRA 900 was proven to be an appropriate resin for phytate extraction and showed significant selectivity toward phytate over other forms of phosphorus [7,8,9]. Our results, therefore, demonstrate the possibility of expanding the knowledge on enabling techniques based on preparative chromatography that may be, given the appropriate studies, taken into consideration for possible pilot and industrial scale-up approaches in an ethanol plant, as the results showed a similar breakthrough profile and a similar breakthrough point range.

A further consideration is that thin stillage is a multicomponent mixture, i.e., more than one species is involved in the adsorption phenomena. In this case, the equilibrium relationship of a single component may not fit a single-component isotherm as competitive adsorption occurs in the overall process. For such a mixture, an ideal adsorbed solution theory can be applied to derive a general equation for multicomponent adsorption isotherms [10]. Among these models, the adaptation of the Langmuir adsorption model to a multicomponent system is presented as Equation (6), in which *q_e,i_* and *q_m_* represent the adsorbate concentration in milligrams per gram for a species *i* at the equilibrium time and the maximum concentration, respectively; *C_e,i_* represents the concentration of species *i* at the liquid phase of the equilibrium (mg L^−1^); *b_i_* is the Langmuir constant (L mg^−1^) for species *i*; and $\overset{n}{\underset{i}{\sum }}\;$is the summation of each species with the *n* species considered in the analysis.

(6) $$q_{e,i}=\frac{q_{m}b_{i}C_{e,i}}{1+\sum_{i}^{n}b_{i}C_{e,i}}$$

The multicomponent Langmuir model was applied to a batch adsorption system using filtered thin stillage as the substrate containing the ions to be adsorbed and IRA 900 as the adsorbent medium. The results presented in Table 1 describe the maximum adsorption capacity of IRA 900 resin on thin stillage regarding NO_3_^−^-N, reactive PO_4_^3−^-P, SO_4_^2−^, as well as phytate-P. The maximum adsorption capacity described herein was derived from the linearization of the multicomponent Langmuir isotherm (data not shown). From this brief analysis, we note that the charge of the anion has a relationship with the maximum adsorption capacity. For the species with the lowest charge, nitrate, the minimum capacity was found; for phytate, which is a species known to have multiple pK_a_ values, the highest numerical values of *q_m_* were obtained. Some additional explanation is given in the Supplementary Material, based on [31,32].

These results demonstrate that IRA 900 resin, likely due to its high charge-to-surface ratio [8], yields a preferential adsorption to anions with a higher charge density. These results are important in demonstrating that phytate is the main ion being adsorbed on the column and likely is the main factor to be taken into account in the scale-up approach.

## 4. Conclusions

In summary, the efficiency of the separation and recovery of a compound in chromatographic-based separations is only dependent upon feed volume, effective plate volume, and the number of theoretical plates. If the feed volume and plate volume are linearly scaled, the number of theoretical plates, not measured per se in this work but demonstrated through breakthrough curves, is an interesting basis for scaling up ion-exchange adsorption or chromatographic columns. Based on previous studies on protein purification, a scale-up model based on the maintenance of the concept of plate numbers was derived for phytate extraction of thin stillage. The results showed a similar breakthrough profile and similar breakthrough point range. Using extrapolation, further considerations can be made for possible pilot and industrial scale-up of this technology.

## Figures and Tables

**Table 1:** Langmuir-derived *q_m_* for phytate-P, NO_3_^−^-N, reactive PO_4_^3−^-P, and SO_4_^2−^.

Anion	*q_m_* (mg g^−1^)	R^2^
Phytate-P	130	0.9206
NO_3_^−^-N	36.28	0.9284
Reactive PO_4_^3−^-P	68.98	0.9345
SO_4_^2−^	47.35	0.9435

## Data Availability

Not applicable.

## Supplementary Materials

The following supporting information can be downloaded at: https://www.mdpi.com/article/10.3390/membranes12020230/s1, Figure S1: (A) Filtration profile of thin stillage under different pressures using a 0.45 μm cellulose filter; (B) Linearization of t/V against V on the filtration of thin stillage; Figure S2: Scheme of Eddy and pore diffusion contributions to axial dispersion on a packed bed (adapted from [22]); Figure S3: Partition chromatography in stages (adapted from [17]); Figure S4: Material balance in a column (adapted from van Deemter et al. [17]); Table S1: Literature data on Cl^−^, NO_3_^−^, and SO_4_^2−^ ion exchange thermodynamic properties. Adapted from [15].
