# Supplementary material for: Volumetric Scale-Up of a Packed-Bed Ion-Exchange System to Extract Phytate from Thin Stillage
Source: Membranes (Basel). 2022 Feb 17;12(2):230. doi: 10.3390/membranes12020230 (PMC8877854; doi:10.3390/membranes12020230)
Supplement: Supplementary file 1 [file membranes-12-00230-s001.zip › membranes-1583941-supplementary.pdf]

membranes

Supplementary Materials

Volumetric Scale-Up of a Packed-Bed Ion-Exchange System to Extract Phytate from Thin Stillage

Cristiano E. Rodrigues Reis [1,2] and Bo Hu [2,*]

1 EARTH University, Las Mercedes, Guácimo, Limón 4442-1000, Costa Rica; crodrigues@earth.ac.cr
2 Department of Bioproducts and Biosystems Engineering, University of Minnesota, Saint Paul, MN 55108, USA
* Correspondence: bhu@umn.edu

Considerations on the filtration of thin stillage

Another factor to be considered considers the preparation of the feedstock material involved in the scale-up approach proposed herein. The particle size analysis of thin stillage details that over 40% of the solids are under a 0.45-μm threshold, while over 90% of the total P is present aggregated or associated with particles smaller than 0.45 μm [6]. In this sense, the presence of unnecessary solids likely would pose additional engineering challenges in the operation of the scalable ion exchange columns. Through a batch filtration of thin stillage, using different pressures, the filtration profile was obtained (Fig. S1A) considering a batch filtration system under different pressures from 10 psi to 50 psi at room temperature, from which, a further analysis can be made based on the linearization of the variables t/V against V (Fig. S1B). The linearization of t/V against V provides an estimation of parameters involved in the scale-up.

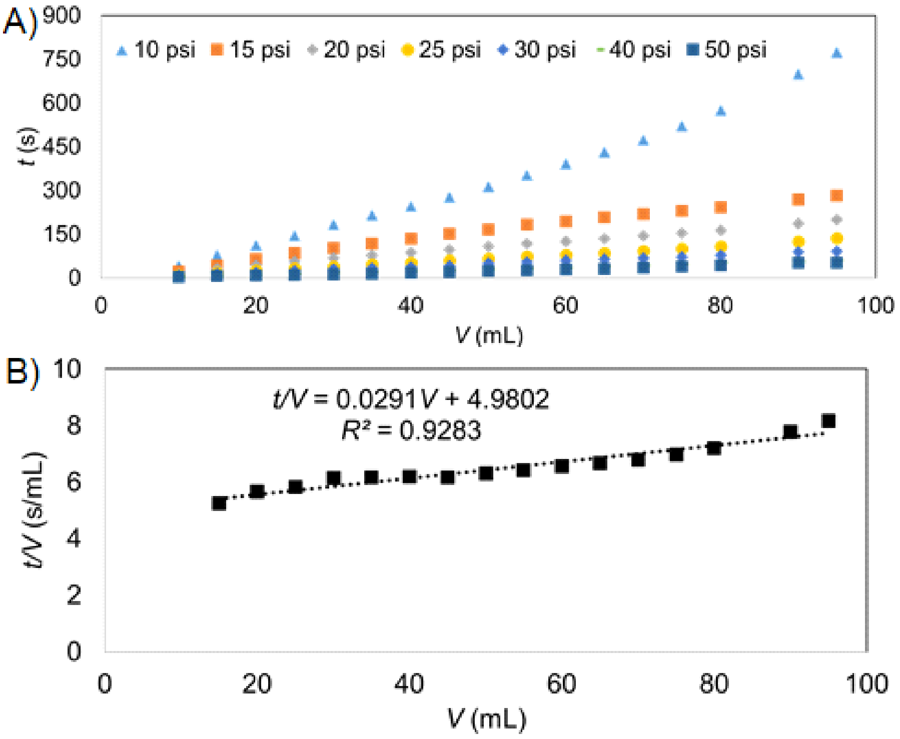

Figure S1. (**A**) Filtration profile of thin stillage under different pressures using a 0.45 μm cellulose filter; (**B**) Linearization of t/V against V on the filtration of thin stillage.

Considerations on the van Deemter model

The van Deemter model describes the mechanisms of band broadening in chromatographic separations for non-ideal separations, i.e., those that do not follow a linear isotherm. It has been described that the rate theory development in systems in which nonideality are caused by axial molecular diffusion, axial Eddy diffusion, and finiteness of transfer coefficient [17]. For a system that follows the two rules of ideal chromatography, in which (i) the equilibrium concentrations are proportional in the two phases, and (ii) the exchange process is reversible according to purely its thermodynamic parameters, i.e., the equilibrium between particle and fluid is immediate, the mass transfer coefficient tends to positive infinity, and there are no diffusion effects within the system, the partition coefficient is related to the ratio of the amounts of those phases present in a column. For ideal chromatography, then, the separation of different solutes can be explained on arithmetic approximations. For systems in which non-linear isotherms are present, i.e., in systems in which the effect of nonlinearity of the isotherm cannot be neglected, usually comprehend conditions with fast mass transfer, and that axial diffusion can be neglected [18], also describing that bands in nonlinear ideal chromatography should be sharp-front and long-tailed.

For non-ideal chromatography and nonlinear isotherms, which comprehend most of the adsorption processes in mixture separations, there exists a comprehensive cooperation of two theories – the rate theory and the plate theory [17]. The plate theory describes the separation efficiency of a chromatographic column by the height equivalent to a theoretical plate (HETP, or simply, H). The rate theory provides all information on the influence of kinetic phenomena and lies its idealization within its acceptability at specific conditions, e.g., thin film cases. This has been applied [19,20] on the analysis of the interpretation of a rate coefficient through a liquid film, as basis [17] to further combine the conditions raised by Glueckeuf et al. [21], who related plate height to particle size, particle diffusion, and diffusion through the film surrounding the particles, especially those regarding Eddy diffusion and intraparticle diffusion. Figure S2 depicts the differences of pore diffusion and Eddy diffusion under such considerations.

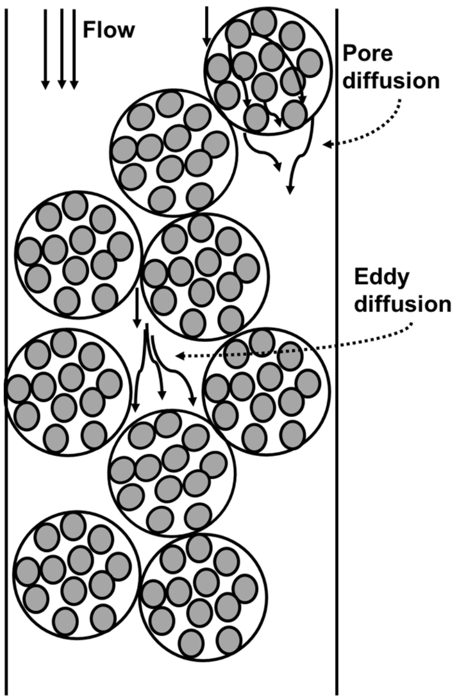

Figure S2. Scheme of Eddy and pore diffusion contributions to axial dispersion on a packed bed (adapted from [22]).

The derivation of the plate theory has been extensively described in the literature [10]. The history and elution curve of a single band in partition chromatography are taken for demonstration, and v_i and v_{ii} are defined as the volumes of the moving and immobile phase in one theoretical plate. For a system with a distribution factor K, assumed to follow a linear profile of concentrations in both phases in the chromatographic separation, i.e., solid, and fluid, the effective plate volume, v, can be thus defined as:

$$v = v_i + \frac{v_{ii}}{K}, \text{ for which, } c_i = Kc_{ii}$$

For a system with feed concentration equals c_0, and the feed volume equals A, the material balance derived for the first plate if a volume dS of the fluid phase passes through is as follows, visually depicted in Figure S3:

$$v_i dc_{i,1} + v_{ii} dc_{ii,1} + c_{i,1} dS = \begin{cases} c_0 dS \text{ for } 0 \le S \le A \\ 0 \text{ for } S > A \end{cases}$$

Similarly for the other plates, i.e., for n>1,

$$v_i dc_{i,n} + v_{ii} dc_{ii,n} + c_{i,n} dS = c_{i,n-1} dS$$

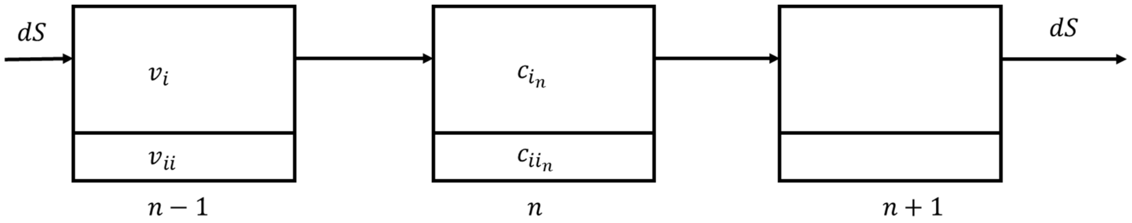

Figure S3. Partition chromatography in stages (adapted from [17]).

The introduction of y_n and w to the equations above, which simplify the concentration volumetric profile and the linear velocity, i.e., $y_n = c_{i,n}/c_0$, and w=S/v, the equations above are simplified to:

$$\frac{dy_1}{dw} + y_1 = \begin{cases} 1 \text{ for } 0 \le w \le \frac{A}{v} \\ 0 \text{ for } w > \frac{A}{v} \end{cases}$$

$$\frac{dy_n}{dw} + y_n = y_{n-1} \ (n > 1)$$

Initial conditions are $y_1 = y_2 = \ldots = y_n = 0$ for w=0.

The solution for all stages can be written as:

$$y_n = \begin{cases} \int_0^w e^{-w'} \frac{w'^{n-1}}{(n-1)!} dw' \text{for } 0 \le w \le \frac{A}{v} \\ \int_{w-\frac{A}{v}}^{w} e^{-w'} \frac{w'^{n-1}}{(n-1)!} dw' \text{for } w > \frac{A}{v} \end{cases}$$

The integrand of y_n for w>A/v is the Poisson distribution function, which can be approximated on conditions in which w is large enough, usually greater than 100, and qualitatively described if the number of plates is not too small (infinitely thin column) or if the band has arrived at the end of the column. Therefore, it can be assumed the following is true:

$$\frac{1}{n!} e^{-w'} w'^n \approx \frac{1}{\sqrt{2\pi w'}} e^{-\frac{(w'-n)^2}{2w'}}$$

For large values of w′, it can be assumed that w′≈n, and that n-1≈n, and redefining w and yn as above, the van Deemter elution curve is described as:

$$c_{i,n} = \frac{c_0}{v\sqrt{2\pi n}} \int_{S-A}^{S} e^{-\frac{(\frac{S'}{v} - n)^2}{2n}} dS'$$

In order to relate width and height of an elution curve to the plate number, van Deemter et al. [17] defined the dimensionless quantities of s and a, as being:

$$s = \frac{S}{v\sqrt{n}} \text{ and } a = \frac{A}{v\sqrt{n}}$$

Which rewrites the equation above as being:

$$\frac{c_{i,n}}{c_0} = \frac{1}{\sqrt{2\pi}} \int_{s-a}^{s} e^{-\frac{1}{2}(s' - \sqrt{n})^2} dS'$$

For the maximum value of $c_{i,n}/c_0$, the equation will be equivalent to an error function, i.e.:

$$\left(\frac{c_{i,n}}{c_0}\right)_{max} = \text{erf}\frac{a}{2\sqrt{2}}$$

Van Deemter et al. [17] also define ΔS as being the width referent to the distance between the points of intersection of the tangents in the inflection points with the horizontal axis. For arithmetic purposes, a new implicit function δ is defined.

$$\delta e^{-\frac{\delta^2}{2}} = (a + \delta) e^{-\frac{1}{2}(a+\delta)^2}$$

Thus,

$$\Delta s = a + 2\delta + \sqrt{2\pi}\frac{a + \delta}{\delta} e^{\frac{\delta^2}{2}} (\text{erf}\frac{a + \delta}{\sqrt{2}} - \text{erf}\frac{\delta}{\sqrt{2}})$$

Despite the mathematical complexity of ΔS, it can be easily seen that both height ($c_{i,n}/c_0$), and width (Δs) are only dependent on a. In summary, the efficiency of separation and recovery of a compound in chromatographic-based separations are only dependent upon the feed volume, the effective plate volume, and the number of theoretical plates.

If a continuous column was to be considered, i.e., the case in which a column does not fully saturate, a mass balance can be written according to the visual representation on figure S4.Lapidus and Amundson [23] described Eddy diffusion present in packed bed behavior, in which the longitudinal diffusion is caused by irregularities in the packing, which provide small signals to the overall diffusion coefficient.

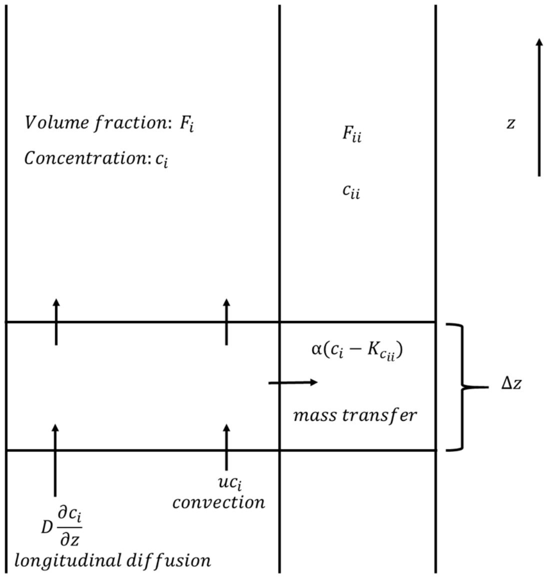

Figure S4. Material balance in a column (adapted from van Deemter et al. [17]).

For a cross sectional area on figure S4, it is seen that:

$$F_i \frac{\partial c_i}{\partial t} = F_i D \frac{\partial^2 c_i}{\partial z^2} - F_i u \frac{\partial c_i}{\partial z} + \alpha(Kc_{ii} - c_i)$$

$$F_{ii} \frac{\partial c_{ii}}{\partial t} = \alpha(c_i - Kc_{ii})$$

Lapidus and Amundson [23] derived the solution for equation above, when a pulse of concentration c_0 during a short time t_0 was introduced to the system. Their solution is read as:

$$\frac{c_i}{c_0} = \frac{zt_0}{2t\sqrt{\pi Dt}} e^{-\frac{z(-ut)^2}{4Dt} - \frac{\alpha t}{F_i}} + \int_0^t \frac{zt_0 e^{-\frac{z(-ut')^2}{4Dt} - \frac{\alpha t}{F_i}}}{2t'\sqrt{\pi Dt'}} \sqrt{\left[\frac{\alpha^2 Kt'}{F_i F_{ii}(t - t')}\right]} e^{-\frac{\alpha K}{F_{ii}}(t-t') - \frac{\alpha t'}{F_i}} I_1 \left\{ 2\sqrt{\frac{\alpha^2 Kt'(t - t')}{F_i F_{ii}}} \right\} dt'$$

The general equation derived from Lapidus and Amundson [23] can be simplified if a column has a large number of transfer units and mixing stages. The number of mixing stages can be defined for a column of height l as ul/2D, in which the height of a mixing stage being equal to 2D/u. The height of a transfer unit is defined by van Deemter as $F_i v/\alpha$.

Considerations on pressure drop

Pressure drop is usually the limit that determines the size of a fixed bed chromatography column, usually being an important parameter to be considered when one is performing a resin screening. The Kozeny-Carman equation describes the pressure drop caused by the friction forces through an incompressible packed resin column:

$$\Delta P = u_0 L \frac{150\mu}{d_p^2}\left[\frac{(1 - \varepsilon)^2}{\varepsilon^3}\right]$$

In which u_0 represents the superficial velocity of the mobile phase, L is the bed height, μ is the viscosity, d_p is the particle diameter, and ε is the void fraction of the column [22]

The Kozeny-Carman equation has limited applications on ion-exchange medium, since most resin material are made from compressible polymeric material (reference). The matrix material in which resins are made of (e.g., polyacrylate) will compress to an extent

depending on the cross-linking, bed height, column diameter, and frictional properties of the medium. The Kozeny-Carman equation also does not account for the presence of a wall material, in which the resin beads are contained. Wall effects are clearly visible on narrow columns, and provide additional support for the column, thus, reducing bed compression. Different geometries will lead to different values of u_{crit}, which is the critical velocity, in which the pressure increases asymptotically, and no additional flow is achieved. Additional flow instability is found in the formation of small and highly compressed regions near the bottom of the column, which represent large deviations from the Kozeny-Carman equation. Colby et al. [24] presented a model to account considering interstitial porosity, specific surface area, internal angle of friction, and the angle of wall friction. The three set of equations presented by [24] are as follows, for frictional support, stress on the particles, and the pressure gradient, respectively:

$$\frac{\partial \tau_{zz}^{s}}{\partial z} = \mu u_0 \chi(\tau_{zz}^{s}) - \frac{4}{d_c} \tan\vartheta \frac{1+\sin\xi}{1-\sin\xi} \tau_{zz}^{s}$$

$$\chi(\tau_{zz}^{s}) = \frac{200\varepsilon_s^2}{d_p^2 \phi^2 \varepsilon_f^3}$$

$$\frac{\partial P}{\partial z} = -\mu u_0\, \chi(\tau_{zz}^{s})$$

In which τ_{zz} represents the stress acting on the resin particles in the axial direction, usually represented in Pa, z is the distance from the inlet of the column, χ (τ_{zz}) being an expression describing the contribution of porosity and specific surface area to the pressure gradient, dc is the column diameter, ϑ being the angle of wall friction, ξ as the internal angle of friction, and ϕ as the shape factor of the particles (m^{-1}).

The solution of these equations is often given by 4th-order Runge-Kutta integration method, and several analyses have reported errors within the 20% range. Soriano et al. [25] presented a column pressure drop model accounting gravity and the forces acting on the bottom, top, and the sides of a differential-sized slice of a column. The semi-empirical model accounts for the constant K from the Blake-Kozeny equation:

$$K = K_0 e^{-\left(\frac{P}{P_{s_0}}\right)}$$

And is written as:

$$\frac{dP_s}{dh} = \Delta\rho(1-\varepsilon)g + K u_0 - P_s k \tan\vartheta \left(\frac{4}{d_c}\right)$$

In which, the new variables are K as the Blake-Kozeny constant, K_0 as the permeability of the uncompressed gel matrix (m^2), P as productivity (g L^{-1} h^{-1}), P_{s0} as the matrix rigidity (kPa), h as the reduced plate height (H/dp), $\Delta\varrho$ as the density difference between the medium and the mobile phase, g as the acceleration due to gravity, k as a pressure coefficient, and P_s as the verticle solid pressure in the bed (kPa).

The most appropriate way to solve the model derived by Soriano et al. [25] is to employ 4th-order Runge-Kutta integration methods, to generate axial pressure-flow curves for a given matrix and column specifications. An empirical model to predict pressure drop from a series of bed compression measurements – using different aspect ratios and column diameters was introduced by Stickel et al. [26]. A linear model is, thus, presented and expects to linearly fit the critical velocities as a function of the aspect ratio; in which m and b are linear regression coefficients.

$$u_{crit} = \frac{m\left(\frac{L_0}{d_c}\right) + b}{L_0}$$

The critical bed compression φ_c can be calculated as being:

$$\varphi_c = \frac{\varepsilon_c - \varepsilon_0}{\varepsilon_c - 1}$$

Which, for any given velocity lower than u_{crit}, the bed compression can be calculated as

$$\varphi = \varphi_c \frac{u}{u_c}$$

For which, m and b are linear coefficient parameters, and L_0 as the initial gravity-settled bed height.

Though pressure drop vs. flow curves are often obtained by the manufacturer, which can be used to estimate the maximum pressure a particular medium can hold, the operation conditions in columns may impose lower limits, especially because of wall effects.

Theoretical and empirical considerations on the competitive anion exchange process

Marcus and Howery [31] presented an equilibrium selectivity ratio, in which z_A and z_B are the charge numbers, [I±] represents the molar scale applied to the solution phase concentration. The mean molar coefficient, $y_{\pm IC}$ is also portrayed in the equation below:

$$k_{b,a} = \frac{\bar{x}_b^{\frac{1}{z_b}} [A_\pm]^{\frac{1}{z_a}} y_{\pm AC}}{\bar{x}_a^{\frac{1}{z_b}} [B_\pm]^{\frac{1}{z_b}} y_{\pm BC}}$$

Considering a constant $k_{b,a}$ as the equilibrium selectivity ratio per equivalent of ions A and B, and x_B as the equivalent fraction of the B ion in the exchanger, a constant k_0 can be calculated as:

$$\log K^0 = \int_0^1 \log k_{b,a} d\bar{x}_b$$

K_0 represents, thus, a dimensionless equilibrium constant for the ion exchange reaction. $-\Delta H^\circ$ represents the negative of the standard integral enthalpy. From a qualitative point of view, a summary of the anions present in thin stillage to which thermodynamic calculations were presented is reported on table. The anions selected herein are those found in thin stillage, to which thermodynamic data was reported in the literature. Though a thorough analysis cannot be done with these results, it can be easily noted that increasing crosslinkage of the resin, an increase in exchange capacity of the ion B is increased, as seen by log K°. Considering IRA 900 is a heavily crosslinked resin [32] with a highly porous surface [6], we assume such factors to be potentially significant in explaining the phytate preference over the other anions. Table S1 presents a summary of similar resin media applied to different exchange systems.

Table S1. Literature data on Cl^-, NO_3^-, and SO_4^{2-} ion exchange thermodynamic properties. Adapted from [15].

Ion A	Ion B	Crosslinkage (%)	Medium	Exchanger	log K°	-ΔH° (kcal)
OH^-	Cl^-	8	NR	$[-R-N(CH_3)_3^+-]_n$	0.38	NR
OH^-	Cl^-	2	NR	$[-R-N(CH_3)_3^+-]_n$	1	NR
OH^-	Cl^-	4	NR	$[-R-N(CH_3)_3^+-]_n$	1.26	1.8
OH^-	Cl^-	10	NR	$[-R-N(CH_3)_3^+-]_n$	1.73	NR
Fl^-	Cl^-	10	NR	$[-R-N(CH_3)_3^+-]_n$	0.9	NR
Fl^-	Cl^-	1	0.1M Na^+	$[-R-N(CH_3)_3^+-]_n$	0.57	1.55
Fl^-	Cl^-		0.1M K^+	$[-R-N(CH_3)_3^+-]_n$	0.43	1.6
Cl^-	NO_3^-	~8	NR	$[-R-N(CH_3)_3^+-]_n$	0.56	0.27
Cl^-	NO_3^-	3	NR	$[-R-N(CH_3)_3^+-]_n$	0.36	NR
Cl^-	1/2 SO_4^{2-}	~8	NR	$[-R-N(CH_3)_3^+-]_n$	0.06	NR
Cl^-	1/2 SO_4^{2-}		NR	$[-R-N(CH_3)_3^+-]_n$	0.02	NR
Cl^-	1/2 SO_4^{2-}		NR	$[-R-N(CH_3)_3^+-]_n$	0.02	-1.1
Cl^-	1/2 SO_4^{2-}		NR	$[-R-N(CH_3)_3^+-]_n$	0.02	-0.92
Fl^-	Cl^-	8	0.01-0.1M Na^+ or K^+	$[-R-N(CH_3)_2(C_2H_4OH)^+-]_n$	1.09	NR
Cl^-	NO_3^-	8	0.01-0.1M Na^+ or K^+	$[-R-N(CH_3)_2(C_2H_4OH)^+-]_n$	0.43	NR
Br^-	NO_3^-	8	0.01-0.1M Na^+ or K^+	NR	0.02	NR

NR: Not reported”
